# TET2-mediated ECM1 hypomethylation promotes the neovascularization in active proliferative diabetic retinopathy

**DOI:** 10.1186/s13148-023-01619-1

**Published:** 2024-01-03

**Authors:** Chunyang Cai, Chufeng Gu, Shuai He, Chunren Meng, Dongwei Lai, Jingfa Zhang, Qinghua Qiu

**Affiliations:** 1 Department of Ophthalmology, Shanghai General Hospital, Shanghai Jiao Tong University School of Medicine, No. 100 Haining Road, Hongkou District, Shanghai, 200080 People’s Republic of China; 2 Department of Ophthalmology, Tong Ren Hospital, Shanghai Jiao Tong University School of Medicine, No. 1111 Xianxia Road, Changning District, Shanghai, 200050 People’s Republic of China; 3 National Clinical Research Center for Eye Diseases, Shanghai Key Laboratory of Ocular Fundus Diseases, Shanghai Engineering Center for Visual Science and Photomedicine, Shanghai Engineering Center for Precise Diagnosis and Treatment of Eye Diseases, Shanghai, People’s Republic of China; 4 Department of Ophthalmology, Shigatse People’s Hospital, Shigatse, Tibet People’s Republic of China

**Keywords:** Proliferative diabetic retinopathy (PDR), Neovascularization, TET2, ECM1, DNA methylation, Epigenetics

## Abstract

**Background:**

Studies have shown that tet methylcytosine dioxygenase 2 (TET2) is highly expressed in diabetic retinopathy (DR), which reduces the DNA methylation of downstream gene promoters and activates the transcription. Abnormally expressed TET2 and downstream genes in a high-glucose environment are associated with retinal capillary leakage and neovascularization. Here, we investigated the downstream genes of TET2 and its potential association with neovascularization in proliferative diabetic retinopathy (PDR).

**Methods:**

GSE60436, GSE57362, and GSE158333 datasets were analyzed to identify TET2-related hypomethylated and upregulated genes in PDR. Gene expression and promoter methylation of these genes under high glucose treatment were verified. Moreover, TET2 knockdown was used to assess its impact on tube formation and migration in human retinal microvascular endothelial cells (HRMECs), as well as its influence on downstream genes.

**Results:**

Our analysis identified three key genes (*PARVB*, *PTPRE*, *ECM1*) that were closely associated with TET2 regulation. High glucose-treated HRMECs exhibited increased expression of TET2 and *ECM1* while decreasing the promoter methylation level of *ECM1*. Subsequently, TET2 knockdown led to decreased migration ability and tube formation function of HRMECs. We further found a decreased expression of *PARVB*, *PTPRE*, and *ECM1*, accompanied by an increase in the promoter methylation of *ECM1*.

**Conclusions:**

Our findings indicate the involvement of dysregulated TET2 expression in neovascularization by regulating the promoter methylation and transcription of downstream genes (notably *ECM1*), eventually leading to PDR. The TET2-induced hypomethylation of downstream gene promoters represents a potential therapeutic target and offers a novel perspective on the mechanism underlying neovascularization in PDR.

**Graphical Abstract:**

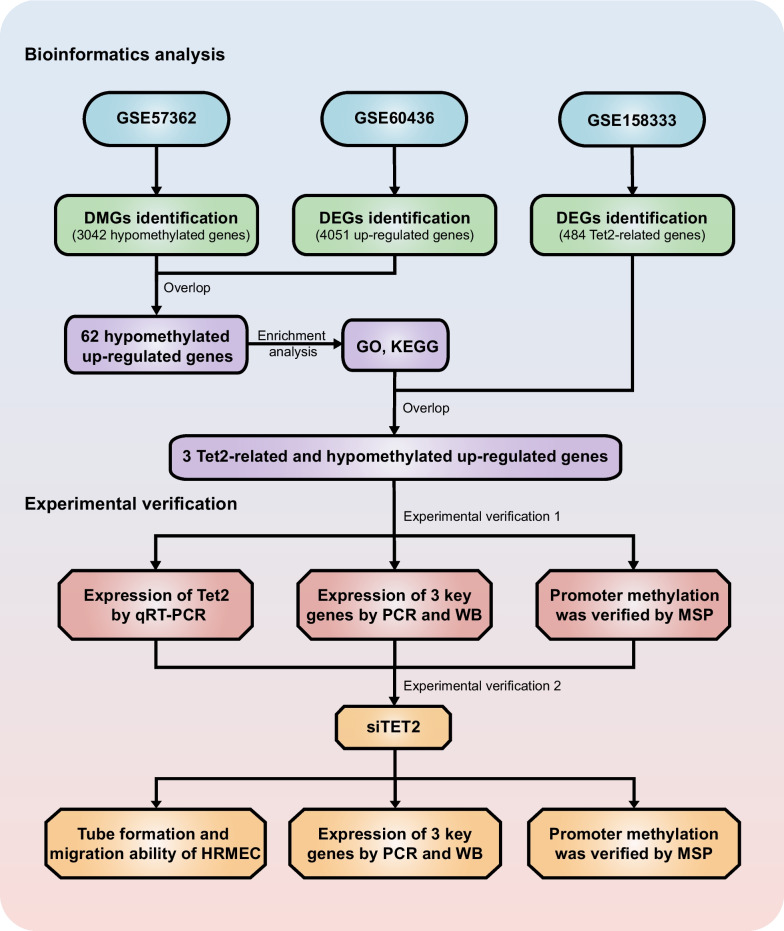

**Supplementary Information:**

The online version contains supplementary material available at 10.1186/s13148-023-01619-1.

## Introduction

Diabetic retinopathy (DR) is a progressive microvascular complication of diabetes mellitus, posing a significant global burden of irreversible visual impairment [1]. With the disease progression, pathological changes such as microaneurysms, intraretinal hemorrhage, and cotton-wool spots gradually appear in the fundus [2]. Finally, the development of retinal neovascularization and fibrovascular membrane (FVM) leads to the proliferative diabetic retinopathy (PDR), with profound complications including blindness [3]. Presently, the clinical treatment of PDR primarily relies on anti-vascular endothelial growth factor (VEGF) therapy, which exhibits limited efficacy in a subset of patients [4]. Therefore, a deeper understanding of the pathological mechanisms underlying PDR is required to identify novel therapeutic targets and interventions.

The development of epigenetics has opened new avenues for investigating the mechanism and treatment of PDR [5, 6]. DNA methylation, a pivotal and widely studied epigenetic mechanism, is involved in the regulation of genome sequence stability and gene expression [7]. CpG hypermethylation at DNA promoter can maintain long-term stable gene silencing by inhibiting transcription factor binding or recruiting chromatin remodeling mediators [8]. In contrast, CpG hypomethylation in gene promoters produces a chromatin state conducive to transcription, facilitating gene expression [8]. The genome, influenced by biological, lifestyle, and environmental factors, can alter gene expression patterns in response to external stimuli by regulating promoter DNA methylation [9]. In various diseases, such as diabetes and cancer, the intrinsic pathological environment can induce adaptive alteration in DNA methylation, subsequently perturbing gene regulation and contributing to disease progression [10, 11]. Notably, abnormal DNA methylation plays a key role in the transformation of DR into PDR, participating in pathological processes such as oxidative stress, inflammation, and neovascularization [12].

Ten-eleven translocation dioxygenases (TETs) are common demethylases that reduce gene promoter DNA methylation levels, thereby stimulating the expression of specific genes [13]. Notably, TETs are activated in the retina and retinal vasculature of patients with diabetes, with tet methylcytosine dioxygenase 2 (TET2) emerging as the prominent isoform [14, 15]. TET2 expression is upregulated in patients with diabetes and enables epigenetic changes, including reduced promoter DNA methylation, through a series of oxidative reaction [15, 16]. Demethylation of promoter DNA alters the protein-DNA interactions and leads to changes in chromatin structure, which promotes the binding of transcription machinery and leads to gene activation [17, 18]. Finally, the abnormal expression of TET2-related genes promotes the development of diabetic complications (such as diabetic nephropathy and DR) by engaging in pathological mechanisms such as oxidative stress and neovascularization [19, 20]. For instance, TET2-mediated reduction of promoter methylation of genes such as matrix metalloproteinase-9 (MMP-9) and ras-related C3 botulinum toxin substrate 1 (Rac1) activates their transcription [15, 21], triggering oxidative stress and mitochondrial damage processes that underlie DR pathogenesis [15, 21]. Moreover, the abnormal overexpression of TET2 in a high glucose environment appears to be associated with retinal capillary leakage and neovascularization [19], emphasizing the potential of TET2-induced hypomethylation of downstream gene promoters as a therapeutic target. Therefore, investigating the role of anti-TET2 strategies and its downstream target genes offers a promising avenue for understanding neovascularization and FVM proliferation in PDR.

In this study, we identified and initially validated three TET2-related genes implicated in the pathogenesis of PDR by analyzing TET2-related gene datasets, gene expression datasets, and gene promoter methylation datasets from the FVM of patients with PDR. Additionally, we elucidated the impact of TET2 on tube formation and migration abilities of vascular endothelial cells and its influence on the expression of these three relevant genes by knocking down TET2. In conclusion, this study provides preliminary insights into the downstream genes regulated by TET2 and sheds light on the potential association of TET2 with the pathological mechanisms of neovascularization in PDR.

## Materials and methods

### Identification of genes from GEO

The Gene Expression Omnibus (GEO; http://www.ncbi.nlm.nih.gov/geo) was employed to acquire three gene sets: (1) set1 “angiogenesis-related genes”; (2) set2 “hypomethylated up-regulated genes”; and (3) set3 “TET2-targeted genes”.

#### Angiogenesis-related genes in PDR

The GSE60436 dataset included gene expression data from three retinal samples from donors without any ocular disease, three active FVM samples from PDR donors, and three inactive FVM samples from PDR donors [22]. This dataset was based on the GPL6884 platform (Illumina HumanWG-6 v3.0). Differentially expressed genes (DEGs) were screened following quality control, normalization, and background correction. The cutoff value of | log_10_ fold change (FC) |> 0.5 and a false discovery rate (FDR) adjusted *P*-value of less than 0.05 were considered statistically different.

“Angiogenesis-related genes” were defined as genes upregulated in active FVM compared with normal retinal samples and downregulated in inactive FVM compared with active FVM. This gene set was generated by intersecting the upregulated genes in the active FVM samples and the downregulated genes in the inactive FVM samples in GSE60436.

#### Hypomethylated up-regulated genes in PDR

The GSE57362 dataset included gene methylation data from 12 retinal samples from donors without any ocular disease and 9 FVM samples from PDR donors [23]. This dataset was based on the GPL13534 platform (Illumina HumanMethylation450). Differentially methylated genes (DMGs) were identified following quality control, normalization, and background correction. |log_10_FC |> 0.5 and an FDR-adjusted *P*-value of less than 0.05 were considered statistically different.

Methylated genes with biological functions were categorized as either “hypermethylated downregulated genes” or “hypomethylated upregulated genes”. For this study, only “hypomethylated upregulated genes” were selected, and these genes were then intersected with gene set1 to obtain common genes. Gene Ontology (GO) and Kyoto Encyclopedia of Genes and Genomes (KEGG) pathway enrichment analyses for these common genes were conducted using the DAVID website (https://david.ncifcrf.gov/). GO terms and KEGG pathways with *P* < 0.05 were considered significantly enriched.

#### TET2-targeted genes

The GSE158333 dataset included gene-expression data from mouse embryonic fibroblasts with wild-type TET2 enzyme (*n* = 3) and mouse embryonic fibroblasts imported with mutant TET2 enzyme (*n* = 3) [24]. This dataset was based on the GPL19057 platform (Illumina NextSeq 500). DEGs were identified following quality control, normalization, and background correction. | log_10_FC |> 0.5, and an FDR-adjusted *P*-value of less than 0.05 was considered statistically significant.

The three obtained gene sets were intersected to obtain “TET2-related hypomethylated upregulated genes in PDR”.

### Cell culture and transfection

Human retinal microvascular endothelial cells (HRMECs) were procured from Applied Cell Biology Research Institute (#ACBRI 181; Kirkland, WA, USA) and cultured in ECM medium supplemented with 5% FBS (#1001, ScienCell, USA) [25]. Cells were treated with normal glucose (NC, 5.5 mmol/L) and high glucose (HG, 30 mol/L) for 48 h [26]. Cultures were incubated at 37℃ in 5% CO2. D-mannitol was used to adjust the osmotic pressure. At the end of the 48-h treatment, cells were digested with trypsin, and DNA or RNA was extracted.

TET2-targeting small interfering RNA (siTET2) and negative control (siNC) were procured from RiboBio (#stB0001257A, RiboBio, Guangzhou, China), while transfection was facilitated using Lipofectamine®RNAiMAX transfection reagent (#13778075, Thermo Fisher Scientific, MA, USA) [27]. Following 8 h of transfection, cells were washed and incubated in 5.5 mM or 30 mM D-glucose medium for 48 h. This resulted in the division of experimental cells into three groups: siNC + NC, siNC + HG, and siTET2 + HG.

### Cell migration assays

The migration ability of HRMECs was determined using a 6.5 mm diameter Transwell system (#3422, Corning, USA). A total of 5 × 10^4^ cells from each group were seeded in the top chamber and cultured in 200 μl serum-free medium, with the bottom chamber containing 650 μl of full serum medium. After 18 h of migration, the migrated cells were fixed with 4% paraformaldehyde for 20 min and stained with 5% crystal violet solution (#A600331-0025, Sangon Biotech, Shanghai, China) for 20 min. Finally, images were acquired using a microscope, and cells were counted in representative areas at 10 × magnification.

### Tube formation assay

The angiogenic capacity of HRMECs was examined by tube formation assay. Each well in a 96-well plate was coated with 10 μl Matrigel (Matrigel Matrix Growth Factor Reduced, #354,230, BD Biosciences, CA, USA) and polymerized at 37℃ for 0.5 h. HRMECs were seeded at 9000 cells per well in 50 μl of complete medium and incubated at 37℃ for a specified duration. The tubular network was imaged with a microscope at 10 × magnification, and the length of the tubular structure was quantified using ImageJ software (NIH, Bethesda, Maryland, USA).

### Protein extraction and western blotting

Proteins extracted from HRMECs were separated by 4–20% SDS-PAGE (#M00930, GenScript, USA) at 140 V for 80 min and transferred onto polyvinylidene fluoride membranes (#IPVH00010, Millipore, Ireland). After blocking with 5% nonfat milk for 2 h, the membranes were incubated with primary antibodies anti-PARVB (#14,463–1-AP, 1:10,000, Proteintech, Wuhan, China), anti-PTPRE (#13,922–1-AP, 1:500, Proteintech, Wuhan, China), anti-ECM1 (#11,521–1-AP, 1:1000, Proteintech, Wuhan, China) or anti-β-actin (#66,009–1-Ig, 1:20,000, Proteintech, Wuhan, China) overnight at 4 °C. After rinsing three times with TBST, the membranes were incubated with a horseradish peroxidase-conjugated secondary antibody (#98,261 and #97,910, 1:50,000, Jackson ImmunoResearch, USA) at room temperature for 1 h. β-actin was used as an internal control. All bands were quantified using ImageJ software, and band densities were normalized to β-actin values.

### RNA extraction and real-time PCR

Total RNA extraction from HRMECs was performed using the EZ-press RNA Purification Kit (#B0004DP, EZBioscience, USA). Reverse transcription to synthesize complementary DNA was performed using the Color Reverse Transcription Kit (#A0010CGQ, EZBioscience, USA). Real-time quantitative PCR (qRT-PCR) was conducted using TB Green™ Premix Ex Taq™ (#RR420A, TaKaRa, Japan). Gene-specific primers were obtained from Sangon Biotech (Shanghai, China), with specific sequences detailed in Table 1. β-actin served as the housekeeping gene (Table 2).

**Table 1:** Real-time PCR primers

Gene	Forward primers	Reverse primers
PARVB	5′-GGAGGTGACGGAACTGGAGAC-3′	5′-AGGTAGAAGTGGTGGAGAGGAAC-3′
PTPRE	5′-GCCGACAGCAACGAGACAAC-3′	5′-AGGAGCACGAGGAGGAGGAG-3′
ECM1	5′-CTGTTGCTTCTGCTGCCTCTG-3′	5′-TCTTCCCTCCTTTCCACTCTGTC-3′
TET2	5′-TCGCAGAAGCAGCAGTGAAGAG-3′	5′-AACTGCCACTGCTGCCACTG-3′
β-actin	5′-GCACCGCAAATGCTTCTA-3′	5′-GGTCTTTACGGATGTCAACG-3′

**Table 2:** Methylation-specific PCR primer

Gene	Sequence	Product size (bp)	PCR conditions
PARVB-M-F	5′-TTTAAAGTGTTGGGATTATAGGTGC-3′	147	95 °C 5 min;
PARVB-M-R	5′-AACAAAAAAAACACAAACAAACGA-3′		
PARVB-U-F	5′-TTTAAAGTGTTGGGATTATAGGTGTG-3′	148	94 °C 20 s,
PARVB-U-R	5′-AAAACAAAAAAAACACAAACAAACA-3′		
PTPRE-M-F	5′-ATTGTTAGTTAGATGGGGGAGGTAC-3′	206	60 °C 30 s,
PTPRE-M-R	5′-ATTGTTAGTTAGATGGGGGAGGTAC-3′		
PTPRE-U-F	5′-ATTGTTAGTTAGATGGGGGAGGTAT-3′	208	72 °C 20 s,
PTPRE-U-R	5′-CCTATAAATAATCCACACCATCATT-3′		
ECM1-M-F	5′-AAATTAGTCGGGTATGGTGGTATAC-3′	132	35 cycles;
ECM1-M-R	5′-TATCGCCTAAAAAAATACAATAACG-3′		
ECM1-U-F	5′-AAAAATTAGTTGGGTATGGTGGTATAT-3′	161	72 °C 5 min
ECM1-U-R	5′-TCACCTAAAAAAATACAATAACACA-3′		

M, methylated; U, unmethylated; F, forward; R, reverse

### DNA extraction, bisulfite conversion, and methylation-specific PCR

Bisulfite transformation of DNA and subsequent methylation-specific PCR (MS-PCR) were performed to quantify DNA methylation differences in the promoter regions of key genes. DNA was extracted from retinal HRMECs using the TIANamp Genomic DNA Kit (#DP304, TIANGEN BIOTECH, Beijing, China), and the bisulfite reaction was performed using DNA Bisulfite Conversion Kit (#DP215, TIANGEN BIOTECH, Beijing, China). The transformed DNA was then eluted with nuclease-free water. MS-PCR reactions of transformed DNA were performed with the MS-PCR Kit (#EM101, TIANGEN BIOTECH, Beijing, China). The reaction system included 2 μL of 10 × PCR reaction buffer, 1.6 μL of 2.5 mM dNTP, 1 μL of each forward and reverse primer, and 1 U of MS-PCR DNA polymerase. Methylation-specific and unmethylation-specific primers for candidate genes were developed using the Methprimer1.0 website (Methprimer1.0; http://www.urogene.org/cgi-bin/methprimer/methprimer.cgi). The resulting MS-PCR reaction products were analyzed on a 2% agarose gel, and Image J software was employed to analyze the intensity of methylated to unmethylated bands (M/U).

### Statistical analysis

Statistical analysis was conducted using SPSS 26.0 statistical software (IBM, Armonk, NY, USA). Cell migration assay was used to count the number of cells per visual field and to quantitatively compare the results between groups. In the tube formation experiment, the tube length of each group was quantitatively counted for statistical analysis. The band densities of agarose gel electrophoresis were quantified by ImageJ software for statistical analysis. Two-tailed Student’s t-test was employed to evaluate statistical differences between NC and HG groups. One-way ANOVA was employed to evaluate statistical differences among three groups (siNC + NC, siNC + HG, and siTET2 + HG). *P* < 0.05 was considered statistically significant. All data are presented as mean ± standard deviation.

## Results

### Identification of TET2-related hypomethylated upregulated genes in PDR

**Fig. 1:**
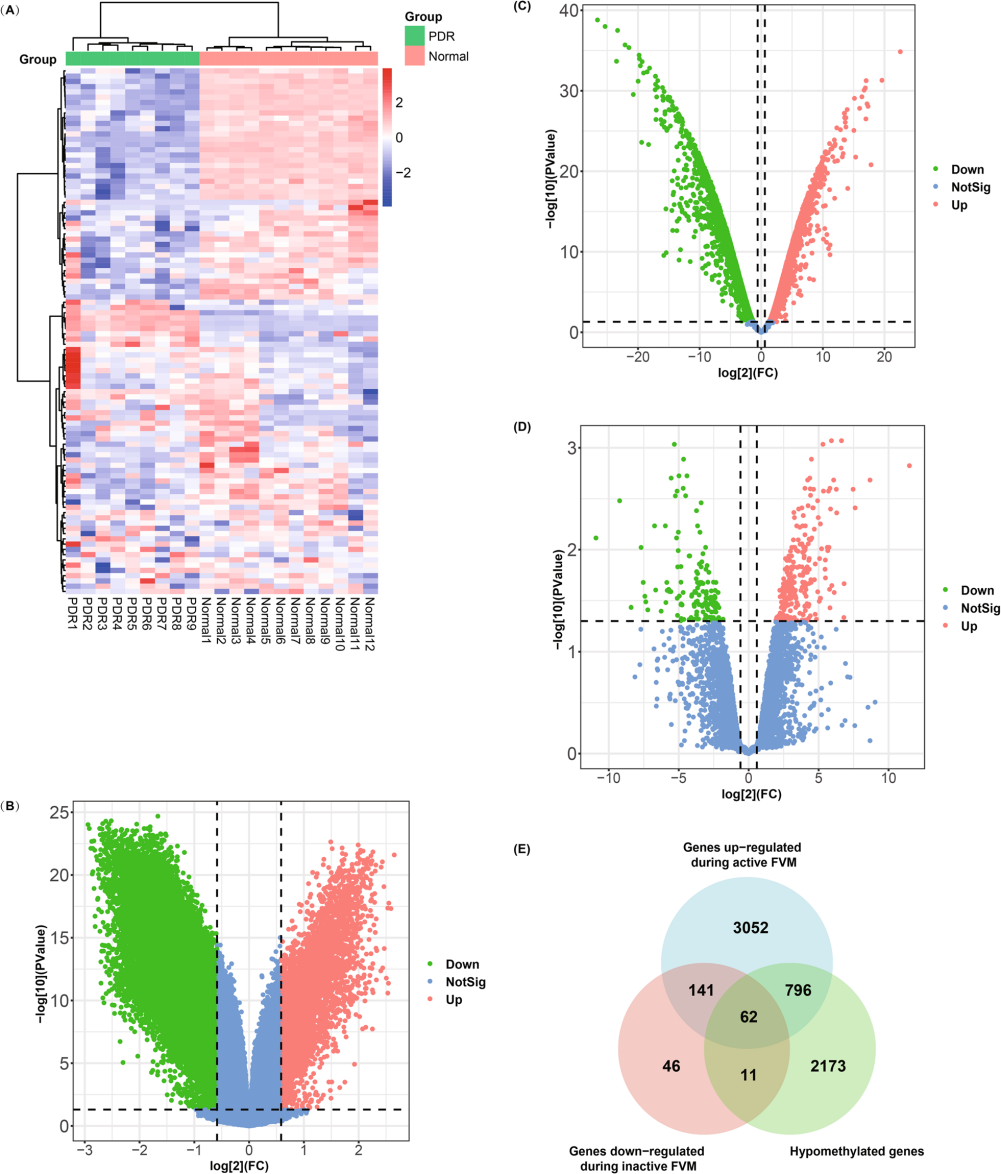
Identification of DMGs. **A** Heat map of the top 100 significant DMGs in the GSE57362 dataset. **B** Volcano plot of the DMGs in the GSE57362 dataset. **C** Volcano plot of the DEGs obtained from the GSE60436 dataset in the active FVM compared with the normal retina. **D** Volcano plot of the DEGs obtained from the GSE60436 dataset comparing the active and inactive FVM. **E** Venn diagram of the intersection of hypomethylated genes, upregulated genes in active FVM, and downregulated genes in inactive FVM. DMGs, differentially methylated genes; DEGs, differentially expressed genes; PDR, proliferative diabetic retinopathy; FVM, fibrovascular membranes

**Fig. 2:**
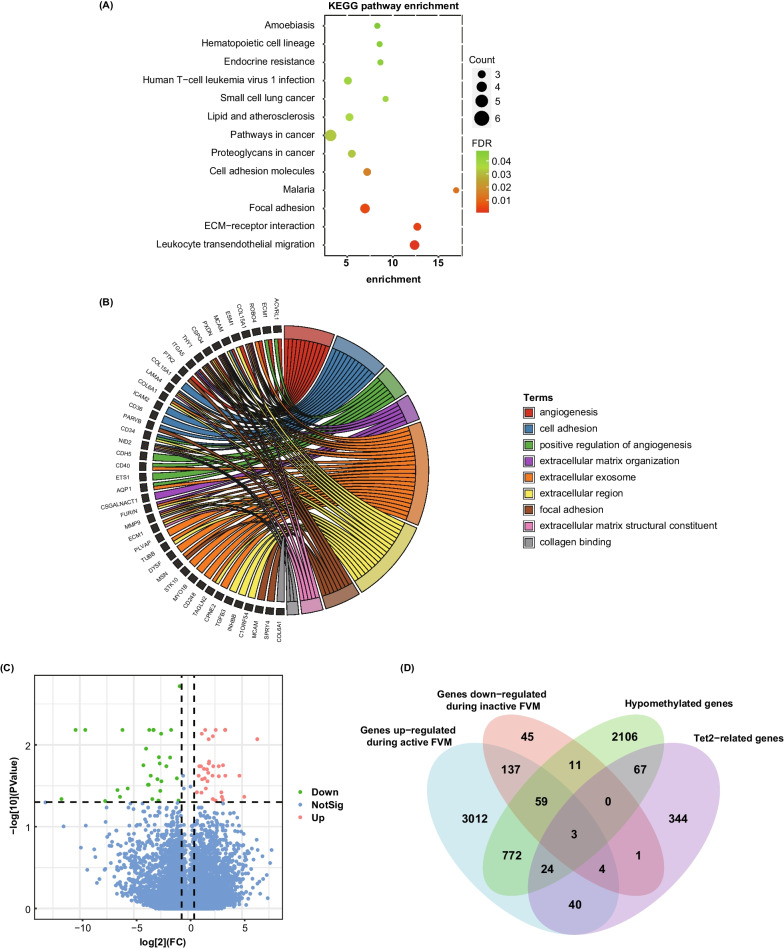
Enrichment analysis of 62 hypomethylated upregulated genes and identification of TET2 downstream target genes. **A** KEGG enrichment analysis of 62 genes. **B** GO analysis of 62 genes. **C** Volcano plot of the DEGs in the GSE158333 dataset. **D** Venn diagram of all gene sets. KEGG, Kyoto Encyclopedia of Genes and Genomes; GO, Gene Ontology; DEGs, differentially expressed genes; FVM, fibrovascular membranes

In the analysis of the GSE57362 dataset, we identified 3,042 genes exhibiting hypomethylation in the context of PDR (Fig. 1A, B). Subsequently, the GSE60436 dataset exhibited 4,051 upregulated genes in active FVM compared with the normal retina, while 260 genes were downregulated in inactive FVM compared with the active FVM (Fig. 1C, D). The above genes were intersected, resulting in the identification of 62 genes exhibiting both hypomethylation and upregulation in active FVM during PDR (Fig. 1E). To gain further insights into the functional relevance of these genes, we conducted GO and KEGG enrichment analysis of 62 genes. The results revealed that these genes were closely associated with angiogenesis, as evidenced by enrichment in pathways such as “angiogenesis” and “positive regulation of angiogenesis” (Fig. 2A, B).

We also utilized the GSE158333 dataset to examine genes targeted by TET2 (Fig. 2C). By taking the intersection of all the gene sets, we identified three TET2-related hypomethylated and upregulated genes: Parvin-β (*PARVB*), receptor type protein tyrosine phosphatase epsilon ε (*PTPRE*), and extracellular matrix protein 1 (*ECM1*) (Fig. 2D). As limited studies have focused on these three genes in diabetes and DR, we carried out further experimental studies on *PARVB*, *PTPRE*, and *ECM1*.

### Methylation and expression levels of key genes following HG treatment

**Fig. 3:**
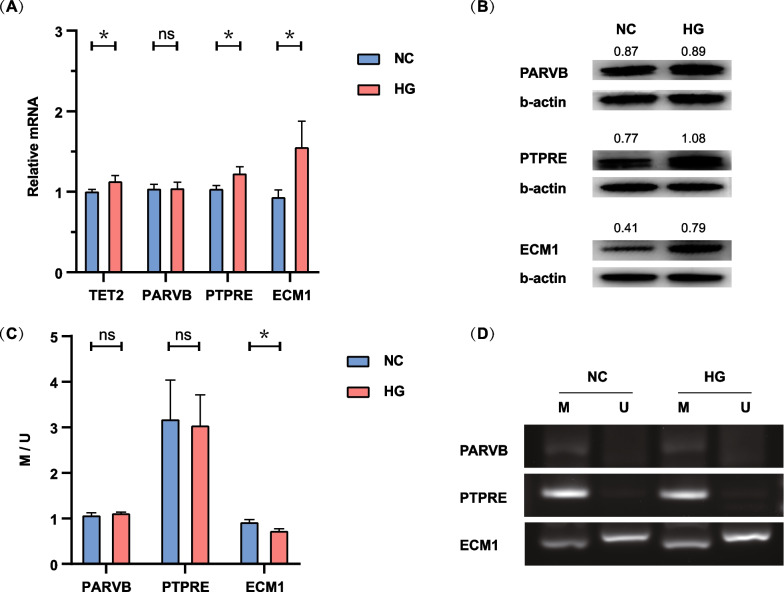
Validation of TET2-targeted hypomethylated upregulated genes. **A** Expression of TET2 and the three key genes. **B** Protein levels of three key genes in NC and HG groups. **C** Quantitative analysis of promoter methylation levels of the three key genes. **D** Agarose gel electrophoresis results of MS-PCR products of the key genes. MS-PCR, methylation-specific PCR; M, methylation bands; U, unmethylated bands; M/U, quantifying the ratio of the intensity of the methylated band to the unmethylated band; NC, normal control; HG, high glucose

Gene expression validation revealed an increase in the TET2 expression in the HG group compared with the NC group. Under high glucose conditions, the mRNA expression of *PTPRE* and *ECM1* was increased, especially *ECM1* (all *P* < 0.05), while *PARVB* expression remained unchanged (*P* = 0.94) (Fig. 3A). The expression changes of the three genes at the protein level were consistent with the expression at the mRNA level (Fig. 3B). Additionally, our validation experiments on the promoter methylation status of the three genes revealed that exposure to high glucose caused a decrease in the methylation level within the promoter region of the *ECM1* gene in HRMECs (*P* = 0.02). However, the promoter methylation of *PARVB* (*P* = 0.31) and *PTPRE* (*P* = 0.84) did not exhibit any significant difference (Fig. 3C, D).

In summary, the following observations were made in HRMECs treated with high glucose: (1) TET2 expression was increased; (2) *PARVB* expression remained largely unchanged, and no significant differences were observed in the methylation status of its gene promoter; (3) *PTPRE* expression increased, along with no significant difference in gene promoter methylation. (4) *ECM1* expression was significantly increased, along with a decrease in the DNA methylation level of the gene promoter. Notably, the degree of promoter methylation was negatively correlated with the gene expression in *ECM1*.

### HRMECs function and key gene expression following TET2 knockdown

**Fig. 4:**
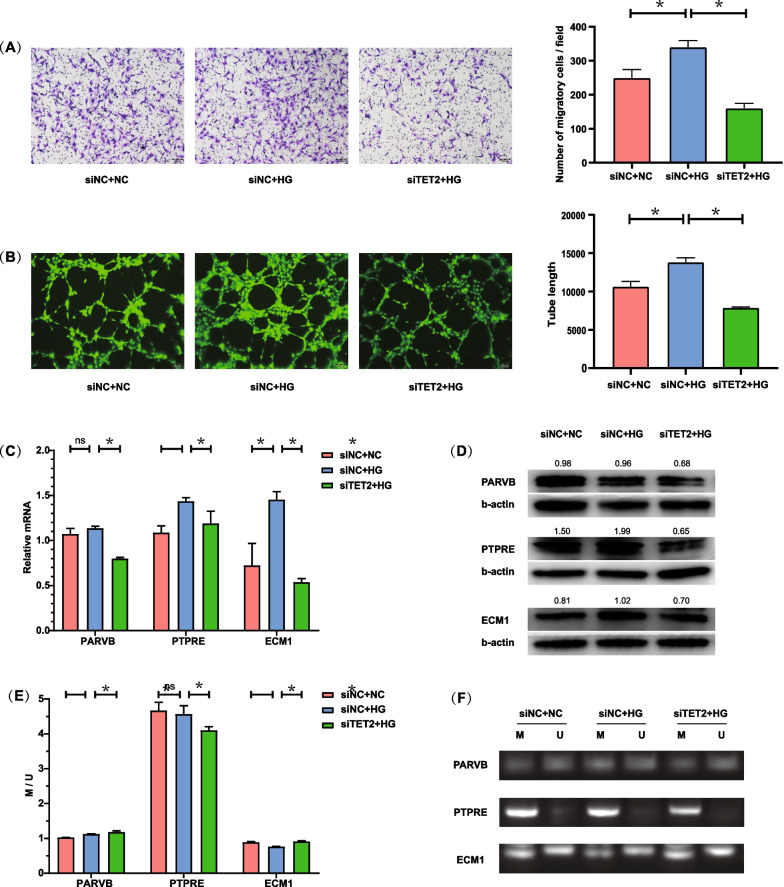
Verification of expression and promoter methylation of the three key genes. **A** Migration assay of HRMECs. **B** Tube formation assay of HRMECs. **C** Quantitative analysis of promoter methylation level of the three key genes. **D** Protein levels of three key genes after TET2 knockdown. **E** Quantitative analysis of promoter methylation levels of the three key genes. **F** Agarose gel electrophoresis results of MS-PCR products of the key genes. MS-PCR, methylation-specific PCR; M, methylation bands; U, unmethylated bands; M/U, quantifying the ratio of the intensity of the methylated band to the unmethylated band; NC, normal control; HG, high glucose

We found a significant decrease in the migration and tube formation ability of HRMECs transfected with siTET2 compared with the control group (all *P* < 0.05) (Fig. 4A, B). Subsequent gene expression verification revealed a reduction in the mRNA expression of *PARVB*, *PTPRE*, and *ECM1* after transfection of siTET2, with *ECM1* showing the most pronounced reduction (all *P* < 0.05) (Fig. 4C). At the protein level, the expression of three genes was decreased after TET2 knockdown, with PTPRE showing the most reduction (Fig. 4D). Furthermore, methylation verification experiments demonstrated that the promoter methylation levels of *PARVB* and *ECM1* in HRMECs increased after TET2 knockdown, particularly in the case of *ECM1* (all *P* < 0.05). However, the promoter methylation of *PTPRE* decreased after TET2 knockdown. (*P* < 0.05) (Fig. 4E, F).

In summary, when TET2 expression was significantly decreased in HRMECs after siTET2 treatment, the following changes were observed: (1) a decrease in *PARVB* expression along with an increase in the methylation level of its gene promoter; (2) a decrease in *PTPRE* expression, with the methylation status of its gene promoter decreasing after TET2 knockdown; (3) a decrease in *ECM1* expression, concomitant with an increase in DNA methylation within the gene promoter region. Notably, promoter methylation of the *ECM1* gene was increased under high glucose but decreased following TET2 knockdown, consistently showing a negative correlation with mRNA and protein expression.

## Discussion

Comprehensive bioinformatics analysis has emerged as an effective method to explore the pathogenesis of various diseases, offering valuable insights into the prediction of biomarkers and therapeutic targets [28]. In this study, we searched three datasets from NCBI-GEO and integrated differences in DNA methylation and mRNA expression between healthy retinal tissues and FVM samples from patients with PDR. Our aim was to delve into the potential role of epigenetic modifications in neovascularization, FVM proliferation, and other pathological alterations associated with PDR. Our findings led to the identification of three “TET2-related hypomethylated upregulated genes”. Experimental verification showed that the expression of TET2 and *ECM1* was significantly increased, while the DNA methylation level of the *ECM1* gene promoter was decreased in HRMECs treated with high glucose. Subsequent TET2 knockdown experiments demonstrated diminished migration and tube formation capabilities of HRMECs. Notably, the expression of *PARVB*, *PTPRE*, and *ECM1*, especially *ECM1*, exhibited a significant decrease following TET2 knockdown, while the methylation level of the *ECM1* gene promoter increased. These findings suggest that TET2 overexpression in a high glucose environment may affect endothelial cell function and contribute to pathological changes such as neovascularization and FVM proliferation, which may be related to the regulation of *ECM1* gene transcription.

DNA methylation has attracted increasing attention in the field of DR. The development of DR is associated with environmental and genetic risk factors, and alterations in epigenetic modifications, such as DNA methylation, may serve as the mechanistic link between environmental exposures and changes in gene expression [21]. The TET family is the most common demethylases, including TET1, TET2 and TET3. Studies on TET1 and TET3 in angiogenesis are mainly concentrated in cancer and ischemic diseases, and its research in diabetic complications, especially DR, is scarce [29–32]. Studies have shown that under the high glucose conditions in patients with diabetes, the oxidative activity of TET2 increases, decreasing promoter methylation in retinal cells. This, in turn, it regulates pathological processes, such as inflammation and oxidative stress, thereby promoting DR development [13, 15]. Additionally, decreased DNA methylation has been identified as a prospective biomarker for patients with PDR [33], such as certain genes in peripheral blood, these include TNF, chitinase 3-like protein 1 (CHI3L1), chimerin 2 (CHN2), and gastric inhibitory polypeptide receptor (GIPR) [34]. Furthermore, the promoter DNA of some specific genes involved in the natural killer cell-mediated cytotoxicity pathway also exhibits hypomethylation in patients with PDR, further indicating that different methylation patterns can be used as non-invasive biomarkers for predicting PDR [34]. Moreover, TET2 can regulate the expression of ROBO4 and its downstream proteins through active demethylation of the ROBO4 promoter, thereby accelerating the development of retinal vasculopathy in diabetes [19]. These findings collectively suggest that TET2-induced hypomethylation of downstream gene promoters is a potential therapeutic target. Strategies involving the modulation of TET2 and its downstream target genes may offer a novel approach for early intervention and the mitigation of DR progression. However, current research on TET2, DNA methylation and PDR is still in its early stages.

**Fig. 5:**
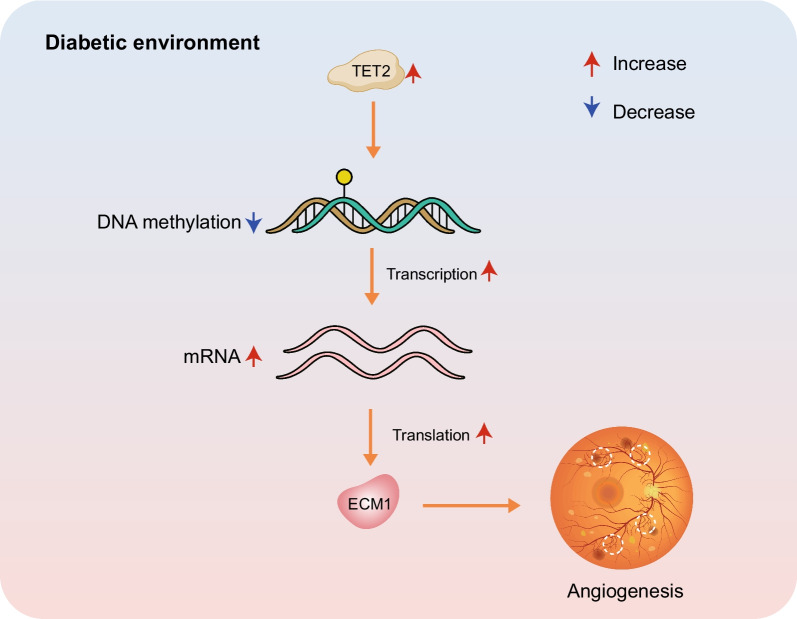
Possible pathological mechanisms of retinal neovascularization and FVM proliferation in patients with PDR. TET2 overexpression in retinal endothelial cells is increased in the diabetic environment, prompting TET2 to act on the CpG island, reducing the methylation level of the *ECM1* gene promoter. The gene of *ECM1* opens and allows transcription, thereby increasing the expression level. Through a series of subsequent pathways, *ECM1* promotes retinal neovascularization and FVM proliferation, ultimately leading to the development of PDR

In this study, we conducted a comprehensive screening and initial verification of three genes downstream of TET2 that may play a role in the pathogenesis of PDR. Among these genes, *ECM1* is an important gene involved in angiogenesis. *ECM1* has been implicated in pathological processes, such as excessive angiogenesis and vasodilation in conditions like psoriasis [35]. Furthermore, its expression is closely related to tumor cell growth, metastasis, and angiogenesis, making it a marker of tumorigenesis associated with aggressiveness and poor prognosis of various cancer types [36, 37]. Interestingly, *ECM1* has also been proposed as a novel tumor suppressor gene, with abnormal hypermethylation of its promoter leading to transcriptional downregulation and contributing to the development of liver cancer [38]. However, to date, no study has explored the relationship between *ECM1* and DR. Our study revealed a decrease in promoter methylation of *ECM1* in HRMECs under high glucose conditions, as confirmed by a reduction in the ratio of methylated to unmethylated DNA promoter (M/U). Upon TET2 knockdown, the methylation level of the *ECM1* promoter increased, accompanied by decreased *ECM1* expression. This suggests that the abnormal expression of *ECM1* in PDR is likely regulated by TET2. Furthermore, enrichment analysis results indicate that *ECM1* may play a role in pathways related to angiogenesis. These findings hint at a possible pathological mechanism in PDR: TET2 overexpression in retinal endothelial cells is increased in the diabetic environment, prompting TET2 to act on the CpG island, reducing the methylation level of the *ECM1* gene promoter. The gene of *ECM1* opens and allows transcription, thereby increasing the expression level. Through a series of subsequent pathways, *ECM1* promotes retinal neovascularization and FVM proliferation, ultimately leading to the development of PDR (Fig. 5).

On the other hand, *PTPRE* is a receptor tyrosine phosphatase with a certain regulatory effect on angiogenesis [39, 40]. *PTPRE* exhibits high expression in vascular endothelium and can inhibit the proliferation of umbilical vein endothelial cells [41]. Abnormal promoter hypermethylation and low expression of *PTPRE* have been linked to cancer development [42], suggesting the protective role of *PTPRE* overexpression. However, in this study, *PTPRE* expression level was significantly increased in HRMECs in the HG group, indicating a potential association with PDR development. This finding may appear contradictory to the protective effect suggested by existing studies. Furthermore, promoter methylation of *PTPRE* exhibited no significant differences between the high glucose and normal group, and the methylation level of *PTPRE* decreased after TET2 knockdown. This suggests that changes in *PTPRE* expression may not be directly associated with abnormal DNA methylation.

Lastly, *PARVB*, a member of the Parvin protein family, plays an important role in cellular processes, such as adhesion, proliferation, and migration [43]. Studies on abnormal *PARVB* promoter methylation have primarily focused on tumor and non-alcoholic fatty liver disease [43, 44]. In tumor studies such as glioblastoma multiforme, hypomethylated and highly expressed *PARVB* may be involved in the process of epithelial to mesenchymal transition (EMT) and significantly associated with poor prognosis, making it a potential target for tumors [43]. Our bioinformatics analysis indicated that epigenetic changes in *PARVB* were closely related to PDR pathogenesis. However, subsequent experiments revealed no significant differences in the methylation of its gene promoter and gene expression between normal and disease states. These findings suggest that *PARVB* may be involved in the angiogenesis and FVM proliferation of PDR through mechanisms that do not involve changes in DNA methylation. Alternatively, it is plausible that *PARVB* may not be an effective gene involved in the pathogenesis of PDR.

This study is the first to apply the datasets to screen for TET2-targeting genes involved in the pathogenesis of angiogenesis in active PDR, which is a way to make full use of research results and save research resources. In this way, we screened and initially validated a potential target. This may be the first study to investigate the association of ECM1 with angiogenesis in PDR. However, our study has some limitations. Based on public databases and previous studies, TET2 is the most critical molecule in DR, so this study mainly focused on TET2 and its downstream hypomethylated genes. Therefore, we cannot exclude the role of other TET family (TET1 or TET3)-targeted genes or hypermethylated downregulated genes in angiogenesis or PDR. Besides, there are no datasets for TET2 knockdown or overexpression in human retinal tissues. In this study, the gene expression dataset of TET2-deficient mouse fibroblasts was selected to analyze the TET2-acting genes. Therefore, not all of the TET2-targeted genes analyzed in this study are applicable to human PDR. This may be the reason why only ECM1 of the three key target genes is more consistent with the regulation of TET2 in PDR.

## Conclusion

In conclusion, this study postulated that TET2, displaying abnormal expression in the diabetic environment, may participate in pathological processes such as neovascularization and FVM proliferation by regulating the methylation and transcription of downstream genes, eventually leading to the development of PDR. Our findings identified *ECM1* as a key potential downstream target of TET2 for its role in DR. While several studies have delved into DNA methylation in the context of DR, investigations into the relationship between abnormal DNA methylation and the pathogenesis of PDR (such as neovascularization and FVM) remain in their infancy. The identification of key target genes affected by DNA methylation and the precise elucidation of their roles in the pathogenesis of PDR warrant further exploration. This study holds the promise of offering a new perspective on the pathological mechanisms of PDR and potential clinical treatment avenues.

### Supplementary Information

**Additional file 1**: List of the top 100 significantly DMGs in GSE57362 dataset.

**Additional file 2**: List of all gene sets. (1) DEGs obtained from the GSE60436 dataset in the active FVM compared with the normal retina; (2) DEGs obtained from the GSE60436 dataset comparing the active and inactive FVM; (3) DMGs in the GSE57362, hypomethylated genes; (4) DEGs in the GSE158333 dataset, TET2-related genes.

## Data Availability

The datasets supporting the conclusions of this article are included within the article (and its additional files).

## Abbreviations

DR: Diabetic retinopathy
FVM: Fibrovascular membrane
PDR: Proliferative diabetic retinopathy
VEGF: Vascular endothelial growth factor
TETs: Ten-eleven translocation dioxygenases
TET2: Tet methylcytosine dioxygenase 2
MMP-9: Matrix metalloproteinase-9
Rac1: Ras-related C3 botulinum toxin substrate 1
GEO: Gene Expression Omnibus
DEGs: Differentially expressed genes
FC: Fold change
FDR: False discovery rate
DMGs: Differentially methylated genes
GO: Gene Ontology
KEGG: Kyoto Encyclopedia of Genes and Genomes
HRMECs: Human retinal microvascular endothelial cells
NC: Normal glucose
HG: High glucose
siTET2: TET2-targeting small interfering RNA
qRT-PCR: Real-time quantitative PCR
MS-PCR: Methylation-specific PCR
PARVB: Parvin-β
PTPRE: Receptor type protein tyrosine phosphatase epsilon ε
ECM1: Extracellular matrix protein 1
CHI3L1: Chitinase 3-like protein 1
CHN2: Chimerin 2
GIPR: Gastric inhibitory polypeptide receptor
EMT: Epithelial to mesenchymal transition
